# Association of Salivary Statherin, Calcium, and Proline-Rich Proteins on Oral Hygiene: A Cross-Sectional Study

**DOI:** 10.1155/2021/1982083

**Published:** 2021-02-23

**Authors:** Deepak Gowda Sadashivappa Pateel, Shilpa Gunjal, Liew Fong Fong, Nur Sulwana Mohd Hanapi

**Affiliations:** ^1^Department of Oral Pathology Oral Medicine, Faculty of Dentistry, MAHSA University, Selangor, Malaysia; ^2^Department of Dental Public Health, Faculty of Dentistry, MAHSA University, Selangor, Malaysia; ^3^Department of Oral Biology and Biomedical Sciences, Faculty of Dentistry, MAHSA University, Selangor, Malaysia

## Abstract

**Background:**

Saliva, as a complex biofluid, plays a pivotal role in maintaining oral health and tooth integrity. There has been inconsistent data available on the relationship between salivary parameters and oral health. This study aims to investigate the association between salivary statherin, acidic proline-rich proteins (aPRP), and calcium with oral hygiene status.

**Methods:**

One hundred and eighty-eight healthy subjects aged between 18 and 50 years with varying oral hygiene status who gave consent to participate were included in this cross-sectional study. The subjects were recruited from primary oral health care of MAHSA University. Oral hygiene of all the participants was measured using Oral Hygiene Index–Simplified (OHI-S). Stimulated saliva collected using paraffin wax was analyzed for salivary statherin, aPRP, and calcium. The relationship between salivary statherin, aPRP, and calcium levels with OHI-S was assessed using Spearman's Rank correlation coefficient; the strength of relationship was assessed by multiple linear regression analysis.

**Results:**

The study found a weak positive correlation (*r* = 0.179, *p* = 0.014) between salivary statherin and OHI-S; weak negative correlation (*r* = −0.187, *p* = 0.010) between salivary aPRP and OHI-S; and moderate negative correlation between salivary statherin and salivary aPRP levels (*r* = −0.50, *p* < 0.001) which were statistically significant.

**Conclusion:**

Poor oral hygiene is associated with increased statherin and reduced aPRP levels in saliva. Thus, these salivary components may have a role in predicting oral hygiene status.

## 1. Introduction

Saliva is a clear, slightly acidic fluid and contains a wide spectrum of inorganic and organic components such as electrolytes, mucus, antibacterial compounds, and enzymes [1]. The complex and diverse nature of salivary composition plays an important role in the maintenance of oral health. In addition, the recent literature reveals that saliva is rapidly gaining importance as a biological fluid of clinical significance. Saliva has the potential to become a first-line diagnostic tool with the help of the advancements made in early detection and the development of biomolecules that have clinical importance [2]. Saliva is composed of a variety of electrolytes, exosomes, microRNA, and cytokines, which are crucial biomarkers in the detection of oral and systemic diseases [3]. As of today, saliva finds a wide range of applications including HIV testing [4], renal disease monitoring [5], chronic kidney disease [6], cardiovascular risk management [7], viral diagnosis [8], forensic medicine, drug abuse monitoring [9], Alzheimer's disease [10], psoriasis [11], stroke [12], and dental studies mainly related to periodontal health and dental caries [13]. Detectable qualitative changes in the salivary proteome could have applications in pathogenesis, diagnosis, and active monitoring of periodontal disease progression and therapeutic monitoring in the future [14].

A group of salivary proteins, statherin, acidic proline-rich glycoprotein, cystatins, and histatins play a vital role in the maintenance of oral health through the regulation of the oral calcium homeostasis by controlling the supersaturated state of saliva with respect to calcium phosphate salts, counter the plaque acidity, formation of dental pellicle, and influence the composition of plaque. These functional proteins prevent the adherence of oral microorganisms and inhibit their growth [15, 16]. A previous study has shown that the salivary statherin and acidic proline-rich proteins are pellicle precursor proteins, due to their strong affinity to hydroxyapatite [17]. These salivary proteins are also believed to play an essential role in regulating the process of bacterial adhesion and could possibly influence the formation of dental calculus [18–20].

Summarizing from the various functions of saliva as mentioned above with respect to oral hygiene, it is interesting to investigate the differences of protective proteins in saliva in various oral hygiene statuses. To date, significant progress has been made in the identification of various salivary proteins and the establishment of their roles in oral health and periodontal disease. However, the roles of salivary statherin and aPRPs are still not clear. Thus, the present study intends to investigate the possible role of salivary statherin, acidic proline-rich protein, and calcium on oral health status.

## 2. Material and Methods

The present cross-sectional study was conducted to correlate the salivary statherin, aPRPs, and calcium levels with the oral hygiene status of the participants. An ethical approval (RP105-10/16) was obtained from the Research Review and Ethics Committee, MAHSA University before subjects' recruitment. A total of 188 healthy volunteer subjects aged between 18 and 50 years were enrolled from primary oral health care of the Faculty of Dentistry, MAHSA University. Informed consent was obtained from each participant before the start of the study. The exclusion criteria include subjects with known systemic diseases like diabetes, under medication with *β*-adrenolytic drugs, salivary gland pathologies, precancerous and cancerous conditions, pathologies of thyroid and parathyroid glands, changes in hormonal profile, and vitamin-D metabolism influencing calcium metabolism, with signs of poor oral hygiene, such as lack of daily teeth-brushing practice and those who have received fluoride treatment within six months of time.

A brief history, oral examination, and demographic data were recorded. Oral hygiene of all the participants was measured by Oral Hygiene Index–Simplified [21]. Before performing the study, intraexaminer reliability was assessed by kappa statistics (0.92). A pilot study was done on 10 subjects to derive the sample size using correlation coefficient. A minimum sample size of 165 was required for “*r*” 0.25, 90% power of the study, and level of significance at 0.05.

### 2.1. Saliva Collection Method

Saliva collection kit (Fitzgerald Industries International, USA) was used for the collection of whole mouth saliva from the study subjects between 10:00 a.m. and 1:00 p.m. All the participants were instructed to fast for 2 hours prior to the collection of saliva. Each participant rinsed their mouth with distilled water for five minutes to remove food debris and followed by chewing paraffin wax to stimulate salivary flow. The stimulated saliva was retained and collected through saliva collection vials; the procedure was repeated until 2 ml of saliva was collected in provided vial bottle. The saliva samples were then stored under −70°C until further analysis. Upon analysis procedure, salivary sample was thawed and centrifuged at 1000 rpm for 20 minutes by using a tabletop centrifuge (Kubota 4000, Japan). The concentrations of statherin, aPRP, and calcium were assessed.

### 2.2. Measurement of Salivary Statherin, Acidic Proline-Rich Protein, and Calcium

#### 2.2.1. Salivary Statherin

Salivary statherin was estimated using ELISA Kit (Elabscience, USA). All the reagents were of analytical grade. Assays were conducted according to the manufacturer's protocol. A standard curve was prepared; standard statherin was centrifuged at 10,000 x g for 1 min; and serial dilution was performed to obtain standard solution 1000, 500, 250, 125, 62.5, 31.25, 15.63, and 0 ng/mL.

Saliva sample (100 *μ*l) was added to a 96-well micro-ELISA plate (dismountable). After incubation at 37°C, 90 min, the liquid was removed from each well and 100 µl of biotinylated detection antibody was added immediately. Then, the ELISA plate was sealed, mixed gently, and followed by 1-hour incubation at 37°C. Then, the plate was rinsed with washing buffer 3 times. Next, 100 *μ*l horseradish peroxidase (HRP) conjugate was added into the wells and mixed gently. After incubation for 30 mins, the plate was rinsed with washing buffer 5 times. A volume of 90 *μ*l of substrate reagent was added to each well and the plate was sealed immediately and incubated for 15 mins. In order to stop the reaction, 50 *μ*l of stop solution was added to each well. The absorbance value of each well was measured at 450 nm.

#### 2.2.2. Salivary Acidic Proline-Rich Protein (aPRP)

Salivary aPRP level was measured using ELISA Kit (Abbexa, United Kingdom). For standard, 100 *μ*l of the diluted standard was pipetted into the standard wells whereas, for control, 100 *μ*l of standard diluent buffer was aliquoted to the control well. In sample wells, 100 *μ*l of centrifuged saliva was added and the plate was shaken to mix thoroughly. After sealing with cover, the plate was incubated for 1 hour at 37°C. After incubation, the liquid was discarded and 100 *μ*l of detection reagent A working solution was added into each well and the plate was incubated for 1 hour. Next, the plate was washed 3 times with wash buffer after the liquid was discarded. The steps were repeated after adding detection reagent B working solution. Then, 90 *μ*l of 3,3′,5,5′-tetramethylbenzidine substrate (TMB Substrate) was added into each well and incubated for 20 mins at 37°C. The reaction was stopped by adding 50 *μ*l of stop solution. The plate was then mixed gently and the absorbance of aPRP was measured at 450 nm immediately.

#### 2.2.3. Salivary Calcium

Calcium level was assessed by using calcium calorimetric assay kit (BioVision, Milpitas, CA, USA). A volume of 25 *μ*l saliva was added into a 96-well plate and topped up with distilled water until 50 *μ*l. For standards, controls, and samples, 90 *μ*l of chromogenic reagent was added. The plate was mixed gently. Then, 60 *μ*l of the calcium assay buffer was added to each well and mixed gently. The plate was then incubated for 10 minutes in the dark at room temperature. The absorbance of the sample was measured at 575 nm.

#### 2.2.4. Statistical Analyses

All assays were performed in triplicate and independently repeated 3 times. The data was analyzed by using SPSS statistical package (version 25.0 SPSS Inc., Chicago, IL, USA). Descriptive statistic was performed to evaluate the distribution and normality of the data. Based on the Kolmogorov–Smirnov test, the normality distribution assumption was checked for quantitative variables. Mean and standard deviation was reported for normal distributed data and median (IQR) for skewed distributed data. Spearman's correlation coefficient was used to find the correlation between salivary statherin, calcium, aPRP, and OHI-S. The significance level was set at *α* = 0.05. Multiple linear regression analysis was used to assess the strength of the relationship between oral hygiene status and salivary proteins.

## 3. Results

The present study is comprised of 188 subjects (87 males and 101 females) with a mean age of 28.6 years. The average value for OHI-S was 1.93 ± 1.09. The median values of salivary statherin level were 33.35 (61.67) ng/ml. The mean salivary aPRP and salivary calcium were found to be 91.52 ± 39.03 ng/ml and 2.86 ± 1.04 mg/ml, respectively, as shown in Table 1.

In a monotonic relationship, the variables tend to change together, but not necessarily at a constant rate. In the present study, Spearman's Rank correlation coefficient was applied to investigate the strength and direction of the relationship between salivary statherin, aPRP, and calcium levels with OHI-S as shown in Table 2. In the present study, a weak positive correlation (*r* = 0.179, *p* = 0.014) between salivary statherin and OHI-S; weak negative correlation (*r* = −0.187, *p* = 0.010) between salivary aPRP and OHI-S; and moderate negative correlation between salivary statherin and salivary PRP levels were observed (*r* = −0.50, *p* < 0.001). However, a correlation between the salivary aPRP and calcium (*r* = −0.03, *p* = 0.686), and salivary calcium and OHI (*r* = −0.137 *p* = 0.060) did not show a statistically significant difference.

There was no interaction amongst the independent variables. No multicollinearity was detected.

Simple and multiple linear regression analysis were performed to know the strength of association between oral hygiene status and salivary proteins. Since the *p* values for both Statherin and aPRP are statistically significant, both variables are included in the multiple linear regression analysis as shown in Table 3. In the current study, the parameter estimate for the “Statherin” variable is 0.001 with a standard deviation of 0.001 and the test statistic *t* = 2.185. For a two-sided test, the probability of interest is 0.03 whereas the parameter estimate for the “aPRP” variable is −0.004 with standard deviation of 0.002 and the test statistic *t* = −2.073. For a two-sided test, the probability of interest is 0.04. The final equation for the analysis is OHI-S = 2.209 + 0.01 (statherin) – 0.04 (aPRP).

## 4. Discussion

Saliva plays a significant role in maintaining periodontal health, helping to build and maintain the health of soft and hard tissues primarily through the formation of dental pellicle selective colonization of nonpathogenic bacteria. Various pathological and protective factors of saliva determine which side the balance swings and whether the oral health problem progresses, reverses, or is in balance [22, 23].

Besides saliva, dental pellicle is another key biological factor that can influence oral hygiene, the mucins, firmly settle on the crystal surface, and create a protective layer. This protective layer of mucous molecules binds water and ions and holds them in place [24, 25]. The noncellular glycoprotein film layer of adsorbed salivary proteins and other macromolecules on the dental enamel surface protects the enamel and serves as a diffusion barrier. It shields the tooth enamel against virulence factors of the oral microbes and facilitates the mineralization process by binding to calcium and phosphates [26, 27]. Salivary proteins such as statherin and aPRPs together with salivary calcium play multiple essential functions towards the maintenance of oral health along with other salivary constituents. Therefore, analysis of upregulation or downregulation of these salivary constituents can lead to better understanding the onset of disease.

The present study demonstrates a weak positive correlation between the level of salivary statherin and OHI-S (*r* = 0.179, *p* = 0.014). The salivary statherin is a potential precursor of dental pellicle, is believed to be the major regulator of mineralization in the mouth, might exert properties [28], and inhibits bacterial adhesion in the dental plaque [29]. Taking into account the results of these studies and other demonstrated protective functions of statherin in the literature including our present study, it may imply that the statherin concentration in saliva could possibly be influenced by the oral hygiene status of the individual and emphasize their protective role in the maintenance of oral health.

The present study has shown a negative correlation of salivary statherin with the calcium levels. This finding is in accordance with our previously reported finding that statherin levels showed a negative correlation with the calcium levels and with calculus formation (14).

On the other hand, a weak negative correlation was found between salivary aPRP and OHI (*r* = −0.187, *p* = 0.01). Acidic PRPs are encoded by two genes, *PRH1* and *PRH2* [30]. It represents 37% of salivary proteins adhered to freshly cleaned teeth and are responsible for various bacterial interactions with the tooth surface [31]. The proline-rich proteins are synthesized by the acinar cells of the salivary glands and their phenotypic expression is under complex genetic control. The aPRPs will bind calcium with a strength which indicates that they may be important in maintaining the concentration of ionic calcium in saliva [32]. Thus, the decrease in the level of aPRP with poorer oral hygiene can be considered as a result of a protective response towards the enamel disruption.

An interesting finding of this project is that a moderate negative correlation between salivary statherin and salivary aPRP levels with a statistically significant difference (*r* = −0.50, *p* ≤ 0.001) was observed. Statherin and aPRPs have many functions in common. They are major contributors of the formation of dental pellicle and play a protective role in maintaining the integrity of the tooth [1]. They promote bacterial adhesion especially *Actinomyces viscosus* [33]. Both statherin and aPRPs have similar roles in the formation of the pellicle covering the tooth enamel and the surfaces of the oral cavity immediately after exposure to saliva [34]. Moreover, the affinity of aPRP and statherin for hydroxyapatite could be different and studies have shown that aPRP has higher affinity than statherin in promoting the attachment of *Actinomyces viscosus* to tooth surfaces in poor oral health condition [35]. These functional differences of the statherin and aPRPs may not clearly explain the significant correlation in the present study. However, the functional similarity of statherin and aPRP could indicate the complementary roles played by them towards each other with respect to their protective functions in the oral cavity. This finding of our study needs to be substantiated through further research.

From the linear regression analysis performed in the study (Table 3), it can be observed that every unit increase in statherin will cause 0.001-unit increase in OHI-S; on the other hand, one-unit increase in aPRP will cause 0.004-unit decrease in OHI-S. Based on the present study, statherin and aPRP can be considered as very weak predictors of oral hygiene status.

The present study has demonstrated that poor oral hygiene is associated with an increased level of statherin which may act as a protective response against enamel disruption. The reduced salivary aPRP may have resulted in calcium precipitation in the plaque leading to lower salivary calcium level in the group with poor oral hygiene. The limitation of the present study encompasses the fact that the subjects were recruited from a single dental school and not from a multicenter, thus limiting the scope of wide generalization.

Aging is an unavoidable part of the life process and the salivary glands are not spared. Aging changes the amount and consistency of the saliva. The physiological functions of saliva gradually deteriorate with the advancing age [36]. Salivary calcium has shown to decrease with the age in healthy individuals [37] and seems to be influenced by many factors especially smoking and periodontitis [38, 39]. Age-related differences in the concentration of salivary PRP's and statherin were demonstrated in young adults compared to children [40] and these proteins were found to be reduced in type 1 diabetic children compared to healthy controls [41]. However, the literature review did not yield any further data for comparison with the older population. The composition of the salivary proteome may have been influenced by the method of estimation, saliva collection method, age, and the severity of the disease. Our study had a wider age group and agewise comparison was not possible. A more complex association involving all the other related salivary proteins with the oral health and disease should be sought. At the same time, it will be interesting to investigate the association of these proteins with other indicators of oral health other than OHI. Further research advents would direct towards greater application of “point-of-care” immunoassays, microfluidics, and protein chip in the management of dental health.

## 5. Conclusion

This study investigated the association between the salivary statherin, aPRP, and calcium with oral hygiene status. Poor oral hygiene is associated with increased salivary statherin and reduced aPRP levels. The salivary proteins like statherin and aPRP may have mutually complementing functions and help in predicting oral hygiene status.

## Figures and Tables

**Table 1:** Distribution of salivary aPRP, calcium, statherin, OHI-S, and demographic characteristics.

	Characteristics	Mean ± SD
Age (years)		28.67 ± 9.3
	Minimum	18^*∗∗∗*^
	Maximum	60^*∗∗∗*^
Gender^*∗*^		
	Males	87 (46.27%)
	Females	101 (53.73%)
OHI-S		1.93 ± 1.09
	Good	67 (35.73%)
	Fair	87 (46.27%)
	Poor	34 (18%)
Salivary statherin^*∗∗*^		33.35(61.67)
Salivary aPRP^*∗∗*^		2.86 ± 1.04
Salivary calcium^*∗∗*^		91.52 ± 39.03

*N* = 188; SD: standard deviation; ^*∗*^number and percent of subjects; ^*∗∗*^salivary statherin and aPRP (ng/ml), salivary calcium (mg/ml), median (interquartile range); ^*∗∗∗*^Number OHI-S Oral Hygiene Index-Simplified aPRP acidic proline-rich proteins.

**Table 2:** Correlation matrix of salivary statherin, aPRP, and calcium levels with OHI.

	OHI-S	Salivary statherin (ng/ml)	Salivary calcium (mg/ml)	Salivary aPRP (ng/ml)
OHI-S				
*r*	1.000	0.179	−0.137	−0.187
*p* value	—	0.014^*∗*^	0.060	0.010^*∗*^
Salivary statherin				
*r*		1.000	−0.104	−0.500
*p* value		—	0.154	<0.001^*∗*^
Salivary calcium				
*r*			1.000	−0.030
*p* value			—	0.686
Salivary aPRP				
*r*				1.000
*p* value				—

OHI-S Oral Hygiene Index-Simplified; r: Spearman's rho correlation; ^*∗*^significant difference between the variables (*p* < 0.05).

**Table 3:** Linear regression analysis.

Independent variable	Simple linear regression		Multiple linear regression	
	b^a^ (95% CI)	*p* value	b^b^ (95% CI)	*p* value
Statherin	0.002 (0.001, 0.003)	0.004	0.001 (0.000, 0.003)	0.03
aPRP	−0.006 (-0.010, −0.002)	0.005	−0.004 (−0.008, 0.000)	0.04
Calcium	−0.114 (-0.265, 0.037)	0.139		

^a^Crude regression coefficient. ^b^Adjusted regression coefficient.

## Data Availability

The data is not published to protect the identity of our participants; however, it can be provided upon request by contacting the corresponding author.
